# An adaptive multi-scale lightweight network for long-distance small traffic sign detection

**DOI:** 10.1038/s41598-026-43920-y

**Published:** 2026-03-13

**Authors:** Ruishi Liang, Wenjie Qu, Shuaibing Li

**Affiliations:** 1 School of Computer Science, University of Electronic Science and Technology of China, Zhongshan Institute, Zhongshan, 528402 China; 2 School of Computer Science and Engineering, University of Electronic Science and Technology of China, Chengdu, 611731 China

**Keywords:** Autonomous driving, Traffic signs detection, Small object detection, Multi-scale lightweight network, You Only Look Once (YOLO), Computational biology and bioinformatics, Engineering, Mathematics and computing

## Abstract

Autonomous driving systems critically rely on the precise detection of distant, small traffic signs to ensure safe and efficient navigation. Nonetheless, existing detection algorithms are confronted with several significant challenges, including the limited efficacy in capturing the subtle visual features of small targets, the adverse effects of complex background clutter, and the imperative for real-time inference via computationally lightweight models. To address these challenges, we propose YOLO-AML, which effectively reduces computational complexity through parameter-free spatial transformations and low-channel convolution operations while preserving fine-grained features of small objects. The proposed Normalization-based Attention with sigmoid and tanh (NAST) module employs a hybrid gating mechanism to precisely regulate attention weight distribution, thereby suppressing background noise without introducing additional convolutional overhead. Furthermore, the C2PSA-LSKA (CLSKA) module integrated into the backbone network enhances the receptive field while minimizing parameter count, effectively mitigating the issue of traffic signs being obscured by background clutter. Additionally, a Normalized Wasserstein Distance (NWD) loss function is introduced to alleviate gradient vanishing commonly encountered with extremely small objects. Experimental results indicate that the optimized model reduces the total number of parameters by 17%, computational complexity by 16.8%, achieves a detection speed of 72.2 FPS, and improves detection accuracy by 2.0%. Grad-CAM heatmap visualization further confirms the model’s enhanced feature discriminability and robustness against background interference. Overall, YOLO-AML demonstrates significant improvements in detection performance under complex real-world driving scenarios.

## Introduction

With the rapid development of autonomous driving technology, traffic sign detection (TSD) has become one of the core tasks of environmental perception systems, directly affecting the safety and reliability of autonomous driving systems^1^. Traffic signs are an important medium for conveying road rules, containing key information such as speed limits, prohibitions, and instructions. Their rapid and accurate detection plays an irreplaceable role in path planning, speed control, and risk avoidance for autonomous driving systems^2^.

In real-world road scenarios, traffic sign detection faces multiple challenges: first, there is a significant variation in target scale, with distant signs accounting for less than 0.1% of the image pixels, while nearby signs may occupy over 10% of the image area^3^; second, environmental interference is complex, with factors such as lighting changes, adverse weather conditions (rain, fog, dust storms), and obstructions (trees, buildings) severely degrading the visual features of signs^4^; third, real-time processing demands strict requirements for the number of parameters and computational complexity. Autonomous driving systems must complete single-frame processing within 50 milliseconds to ensure decision-making latency remains within safe limits^5^, and onboard terminals are typically constrained by computational resources, imposing stringent requirements for algorithm lightweighting^6^.

The development of traffic sign detection technology can be divided into two stages: traditional computer vision methods, two-stage methods based on deep learning, and one-stage methods. Traditional traffic sign detection methods rely on a combination of manually designed features (such as HOG, SIFT) and shallow classifiers (SVM, random forest)^7^. While these methods have low computational complexity, they exhibit poor generalization capabilities in complex scenarios.

One-stage detection algorithms, represented by the YOLO series, achieve a balance between accuracy and speed through an end-to-end regression strategy. Versions such as YOLOv4^8^, YOLOv7^9^, and YOLOv8^10^ have demonstrated outstanding performance in traffic sign detection tasks, but they still have two limitations: first, the contradiction between lightweight design and high accuracy; second, insufficient adaptability to complex scenes. The newly released YOLOv11^11^ has optimized its backbone structure and loss function, but its default configuration has not been specifically designed for the fine-grained features of traffic signs, and its deployment performance on resource-constrained devices remains to be improved.

This paper proposes a lightweight, high-precision traffic sign detection algorithm based on the YOLOv11 framework. The objective of this proposal is to achieve real-time, efficient detection on resource-constrained devices. The innovative contributions of this paper are as follows:

1. In our network, we abandon the traditional convolution approach. Instead, we use a parameter-free spatial-to-depth transformation to fully transfer fine-grained information to the channel dimension. This significantly reduces computational load and memory usage while effectively improving the accuracy of detecting small objects.
2. To address the issue of traffic signs being obscured by background noise, we propose a Normalization-based Attention with sigmoid and tanh(NAST) module with a hybrid gating mechanism that controls the distribution of attention weights more finely, enhancing the model’s ability to distinguish between feature channels.
3. This paper proposes the C2PSA-LSKA(CLSKA) module, which combines LSAK with C2PSA to provide location-sensitive, dynamic weight distribution. The CLSKA module replaces the traditional LSAK’s two-dimensional large kernel convolution with cascaded depth-separable convolution. This expansion of the effective receptive field is achieved while reducing the number of parameters, resulting in a lightweight design without performance loss.
4. The Normalized Wasserstein Distance(NWD) loss function is integrated into the network, whereby the bounding boxes of small traffic signs are modeled as two-dimensional(2D) Gaussian distributions. The traditional IoU metric is replaced with the Wasserstein distance. This effectively addresses the issue of gradient vanishing caused by IoU’s extreme sensitivity to small shifts in target position. This property of NWD has been demonstrated to lead to a substantial enhancement in label assignment and regression accuracy for targets of small size.

The remainder of this paper is structured as follows: Section “Related work” provides a comprehensive review of traffic sign detection-related research, encompassing conventional methods, deep learning-based approaches, and the most recent advancements in the field. Section “Method” delineates the comprehensive framework of the enhanced traffic sign detection algorithm, encompassing the design of the lightweight backbone, adaptive feature module, and loss function. Section “Experiments” introduces the experimental environment and dataset, and validates the efficacy of the proposed algorithm through experimental verification and analysis. Section “Conclusion” offers a synopsis of the aforementioned research and explores prospective directions for future studies.

## Related work

The iterative evolution of autonomous driving technology is contingent upon the efficacy of traffic sign detection and recognition tasks. As one of the core tasks in the autonomous driving perception chain, the accuracy and robustness of feature extraction in traffic sign detection and recognition directly determine the reliability of subsequent decision-making modules. This role is of paramount importance. Existing detection methods can be categorized into two types. The first type is traditional machine learning-based manual feature extraction methods. These methods primarily rely on the physical characteristics of traffic signs, such as color^12,13^, shape^14,15^, and edges^16^. The second type is deep learning-based methods centered on convolutional neural networks.

### Conventional methodologies

Preliminary studies on the automated detection of traffic signs have predominantly relied on manually designed low-level visual features. These features are characterized by the explicit modeling of color, geometric, and texture cues, which are employed to generate and classify potential regions of interest. Li et al.^17^ developed a color probability model in the Ohta color space, generated a probability map, and extracted contour information to locate signs. Nguwi and Kouzani^18^ adopted a similar strategy in the HSI space, employing H-component interval filtering to enhance robustness to changes in sunlight angle. They completed candidate region category discrimination with a three-layer multi-layer perceptron (MLP).

At the edge level, Salti et al.^19^ further introduced HOG (Histograms of Oriented Gradients) features, cascaded with a linear SVM, to achieve sign recognition in nighttime scenes through local texture statistics using 8$\times$8 cells and 9 directional bins. Wang et al.^20^ also combined HOG features with an SVM classifier in the German Traffic Sign Detection Competition, achieving excellent performance; Reference^21^ combined BCNN with HOG features to perform traffic sign detection on the GTSDB^22^ benchmark dataset. However, these hand-crafted features primarily rely on shallow color or edge cues, making them highly susceptible to illumination variations, particularly for distant traffic signs. To address this, the proposed SPD-Conv module (Section 3.1) rearranges, rather than discards, pixels during downsampling, thereby transferring fine-grained textual information into the channel dimension without increasing parameters. In contrast to earlier methods based on low-level cues, the SPD-Conv ensures lossless resolution reduction and, to the best of our knowledge, is the first to apply this technique to traffic-sign detection.

### Deep learning-based approach

The evolution of deep learning paradigms has profoundly reshaped the field of object detection, driving a shift from traditional manual feature extraction to end-to-end representation learning paradigms. Among these, one-stage detection algorithms, exemplified by SSD^23^ and the YOLO series, and two-stage detection algorithms, represented by the R-CNN series^24–27^, have become the mainstream directions. Natarajan et al.^28^ proposed a lightweight weighted parallel CNN network that improves detection speed through a parallel convolutional structure, but it fails to effectively solve the problems of false positives and false negatives of traffic signs under conditions of deformation, fading, or low light. To improve accuracy, Cai et al.^29^ proposed the Cascade R-CNN framework, which adopts a cascaded multi-level detector structure and optimizes detection box quality by progressively increasing the IoU threshold, enhancing the model’s adaptability to complex objects. However, its ability to fuse multi-scale features remains insufficient.

Although these two-stage models exhibit outstanding accuracy, they lag behind in terms of training time and detection speed. In contrast, one-stage detection algorithms offer high speed, making them particularly suitable for mobile applications and aligning with the current development needs in the autonomous driving context. One-stage detection algorithms are widely adopted for their fast detection speed and real-time detection capabilities, especially the YOLO series of algorithms. Han et al.^5^ proposed EDN-YOLO for multi-scale traffic sign detection tasks in complex scenes, using EfficientViT as the backbone network to enhance global feature perception capabilities; by decoupling the detection head to separately handle classification and regression tasks, and combining CIoU and NWD joint loss to enhance sensitivity to small objects, they significantly improved the detection performance of multi-scale traffic signs. Qian et al.^4^ proposed the TSDet method, embedding Swin-Transformer into the CSP module of YOLOv5 (CSP-SwinT), and using window attention mechanisms to capture contextual information of small-scale traffic signs. Zhang et al.^30^ designed the YOLO-BS algorithm, integrating a bidirectional feature pyramid network (BiFPN) and a small object detection layer into YOLOv8. By enhancing multi-scale information interaction through weighted feature fusion, they validated the significant benefits of BiFPN for small object detection. Zheng et al.^31^ proposed the YOLOv8-ALWP algorithm, which optimizes feature extraction by introducing an adaptive downsampling module (ADown) and a separable large kernel attention mechanism (LSKA), while using the Wise-Focaler-EIoU loss function to improve small object detection accuracy. Although cascade detectors raise box quality, the progressively higher IoU thresholds dramatically reduce positive samples of tiny signs, leading to gradient vanishing. To this end, Section 3.4 replaces CIoU entirely with Normalized Wasserstein Distance (NWD) loss, modeling each box as a two-dimensional(2D) Gaussian. NWD provides non-zero gradients even when the predicted box has zero overlap with the ground truth.

### Model lightweighting techniques

Traffic sign detection is a core task of intelligent transportation systems, providing real-time environmental information for autonomous driving. However, scenarios such as in-vehicle terminals and roadside edge devices are subject to hardware constraints such as limited computing power, tight memory, and high real-time requirements. Therefore, model lightweighting has become a key factor in the practical application of this technology. Lightweight networks aim to minimize the number of parameters and computational complexity while maintaining model accuracy, thereby improving network speed and achieving a balance between lightweight design, real-time performance, and accuracy, making them suitable for resource-constrained devices. In 2021, Tan and Le proposed EfficientNetV2^32^, which uses Fused-MBConv and adaptive regularization strategies to increase training speed by 5–11 times while maintaining parameter efficiency. Lau et al.^33^ decomposed the k$\times$k deep convolutional layer in the LKA module into cascaded 1$\times$k and k$\times$1 separable convolutions, proposing the LSKA structure; while maintaining the same accuracy as VAN, the parameter counts of the Tiny/Base models decreased by 2.5%/1.5%, FLOPs decreased by 11.1%/2%, and inference speed improved by 7.6%/3.9%, achieving lightweight and efficient large-core attention networks.

Currently, in the field of autonomous driving, numerous traffic sign detection algorithms based on YOLO have emerged. Breakthroughs in the lightweight model design and embedded deployment technology of the YOLO series algorithms have also provided practical solutions for resource-constrained in-vehicle devices. Sunkara et al.^34^ replaced all stride convolutions and poolings with Space-to-Depth (SPD) transformations to construct the SPD-Conv module; on YOLOv5-SPD and ResNet-SPD, the small object APS improved by 6.7%–19%, while the number of parameters and latency remained largely unchanged, successfully providing a lightweight downsampling scheme with no information loss for small objects and low-resolution scenes. Fang et al.^3^ proposed the YOLO-ADual model, which introduces the C3Dual module and Adown downsampling unit based on the YOLOv5s framework, combined with the CBAM attention mechanism to focus on small object features, balancing deployment efficiency on mobile devices with detection accuracy. Each step in the development of lightweight networks has significantly improved network efficiency and performance on edge devices, laying a solid foundation for mobile and embedded vision applications. In the quest for simultaneously boosting tiny-object accuracy and shrinking model size, Zhou et al.^35^ introduced VDTNet, whose “SPPS-ResNeck-attention” co-design realizes real-time ground-to-air drone detection with only 3.9 MB parameters; its compress-then-compensate strategy offers a ready-to-use paradigm for long-range small traffic-sign detection. Yang et al.^36^ designed ETS-YOLO using the C3Efficient module, DySample upsampling, and NWD loss, further improving the accuracy and real-time performance of small traffic sign detection while maintaining light weight. Prior works often prioritize computational efficiency through channel compression or depth-wise convolutions, which ultimately compromise the expansive receptive fields critical for detecting tiny objects. Our CLSKA module (Section 3.2) first incorporates LSKA large separable kernels into the parallel branches of C2PSA, expanding the receptive field and achieving an inverse balance of larger receptive field vs. fewer parameters.

### Strategies of small object detection

Addressing the issue of IoU being extremely sensitive to small object position shifts, Wang et al.^37^ proposed Normalized Wasserstein Distance (NWD), modeling bounding boxes as two-dimensional(2D) Gaussian distributions to provide stable gradients for traffic signs. In terms of attention, Liu et al.^38^ utilized batch normalization scaling factors to suppress insignificant weights, thereby strengthening the channel and spatial attention for small objects with minimal computational overhead, providing an efficient and lightweight feature re-calibration scheme for small object detection. Meanwhile, during the training phase, the extremely imbalanced foreground-background ratio makes the network prone to being dominated by easy-to-classify negative examples. In terms of small object detection in YOLO, Du et al.^6^ proposed TSD-YOLO, an improvement over YOLOv8s, to address the issue of small traffic signs being easily missed. They introduced a space-depth (SPD) module to expand the receptive field and retain detail information, improving small object detection accuracy while maintaining real-time performance. To alleviate information loss of small objects during downsampling, Zhou et al.^39^ recently proposed DEANet, which employs a Downsampling-Enhancement Module and a parameterized activation function (ACLU) to significantly boost surface-anomaly detection accuracy while maintaining only 2.8 M parameters, offering valuable insights for long-range small traffic-sign detection. Wang et al.^40^ proposed the S-YOLOv11 model to address the issue of small object feature loss in drone images. They designed an enhanced multi-branch auxiliary feature pyramid network (EMAFPN) and a shared detail enhancement detection head (ESDCDH). For high-accuracy tiny-object detection under extreme memory constraints, Zhou et al.^41^ introduced DRNet, which achieves heavyweight-level accuracy on 1280$\times$1280 images with only 309 kB parameters; its synergistic “compress-attend-fuse” paradigm offers a ready-to-use lightweight blueprint for long-distance small traffic-sign detection. Although NWD was proposed for tiny objects, its integration with the YOLO family has rarely been explored, and no mechanism suppresses background clutter. In our work, the standalone NAST module (Section 3.3) first leverages BN scaling factors to mask noisy channels at zero parameter cost, boosting signal-to-noise ratio. Subsequently, the NWD loss (Section 3.4) computes the Wasserstein distance on the cleaned features. The two modules operate sequentially: NAST denoises and NWD refines, raising mAP@0.5 from 82.4% to 84.7% while increasing FPS by 72.2. This noise-suppression first, distribution-regression second pipeline better suits real-world driving scenes.

## Method

**Fig. 1:**
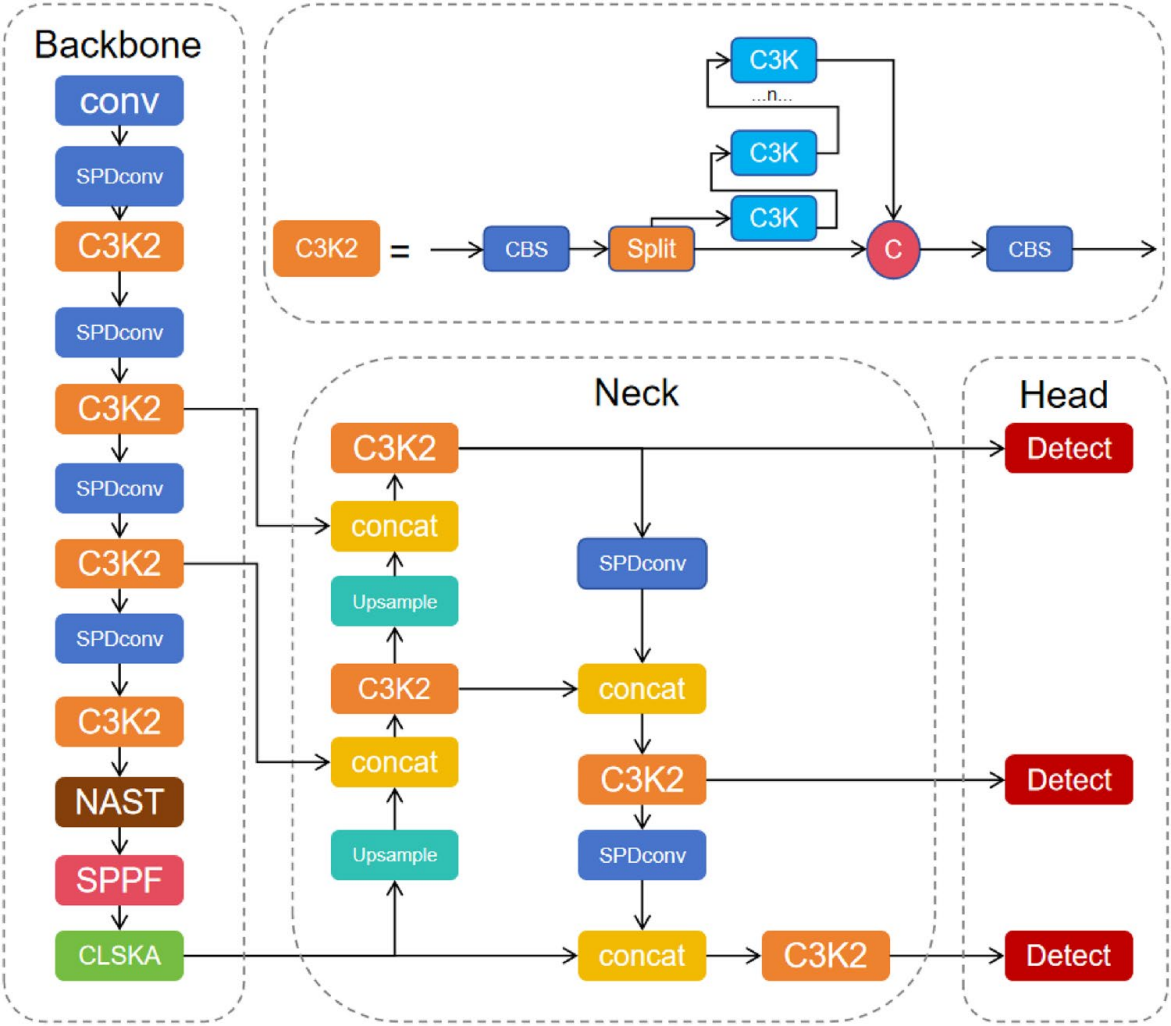
YOLO-AML network structure diagram.

To address the real-time safety requirements of autonomous driving, this chapter introduces an adaptive multi-scale lightweight network framework for detecting small traffic signs at long distances. It uses YOLOv11 as a baseline and is designed around the dual objectives of “high compression ratio” and “high small object accuracy”. The overall architecture is shown in Fig. 1.

### SPD-Conv instead of standard convolution

**Fig. 2:**
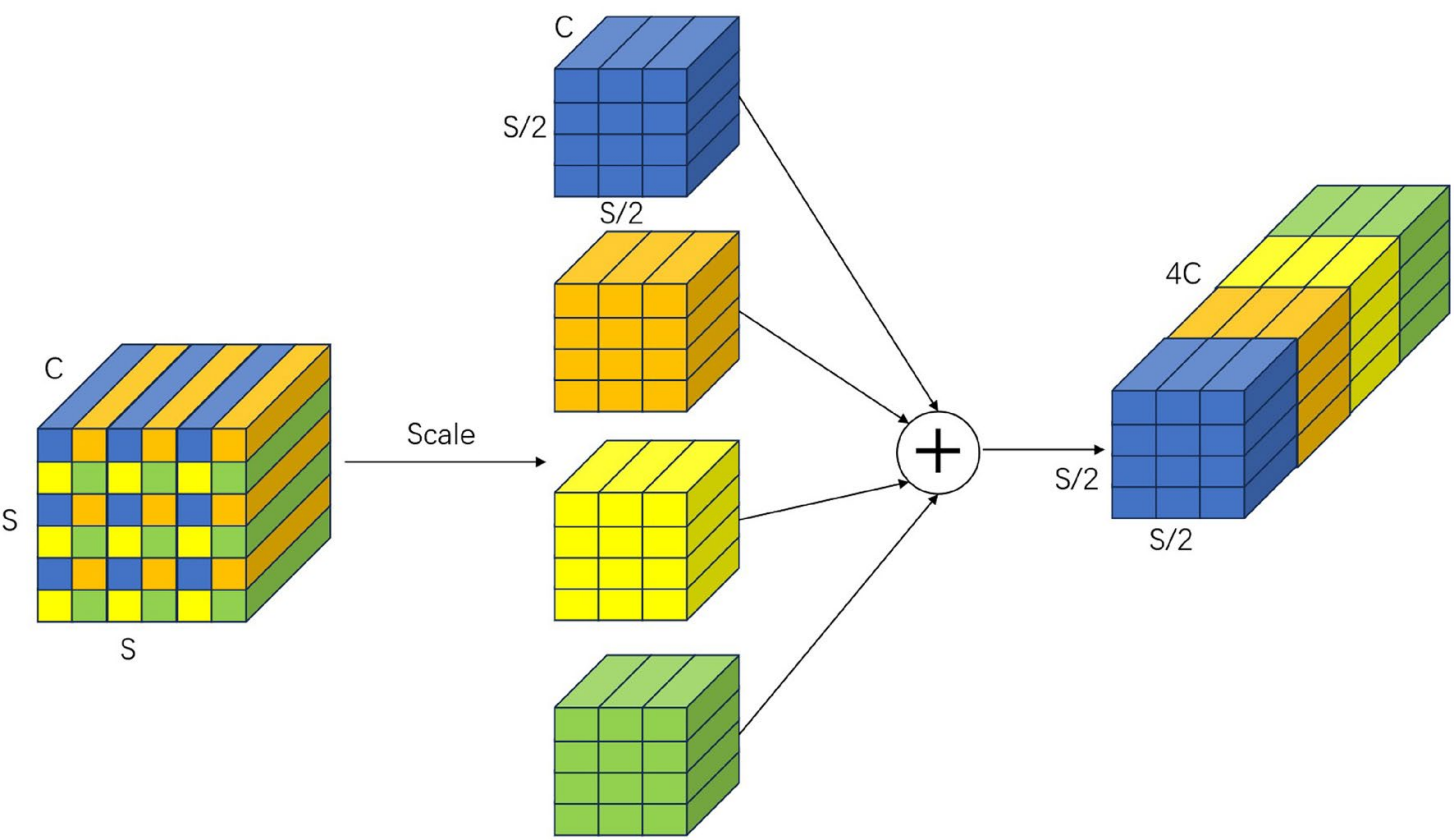
SPD-Conv when scale = 2.

Deep convolutional networks often use consecutive strided convolutions or pooling to quickly reduce the size of feature maps when extracting features of small objects such as traffic signs, thereby reducing the overall computational burden. Input images are often enlarged to 640$\times$640 or higher to preserve sufficient spatial resolution for signs that only occupy a dozen or so pixels. However, this “large-step” sampling immediately discards a significant amount of fine-grained information, and once lost, this information cannot be recovered by subsequent layers, preventing them from retrieving the discarded fine-scale structures. SPD-Conv^34^ is a lightweight alternative unit designed to address this shortcoming. By retaining all original pixels and reorganizing them along the channel dimension, SPD-Conv achieves “lossless downsampling” without increasing the number of parameters. Its lightweight nature and information retention mechanism that is friendly to small objects perfectly align with the dual requirements of lightweight and high precision in traffic sign detection. Figure 2 illustrates the working mechanism of SPDConv when scale=2. And the overall changes in SPDConv are summarized in Equation (1).

The input tensor *X*(*S*, *S*, *C*) is sliced into $\text {scale} \times \text {scale}$ sub-maps of size $(S/\text {scale}) \times (S/\text {scale}) \times C$; these are concatenated along the channel axis to produce an interim map $X'$ whose channel count becomes $C_1 = C \times \text {scale}^2$; a stride-1 convolution then reduces the channels to $C_2$ and yields the output.

1 $$\begin{aligned} X(S, S, C)^{\textrm{SPD}} \rightarrow X^{\prime }\left( \frac{S}{\textrm{scale}}, \frac{S}{\textrm{scale}}, \textrm{scale}^{2} C_{1}\right) {\mathop {\rightarrow }\limits ^{ \text{ Conv } }} X^{\prime \prime }\left( \frac{S}{\textrm{scale}}, \frac{S}{\textrm{scale}}, C_{2}\right) \end{aligned}$$

### Improved C2PSA module with LSKA

**Fig. 3:**
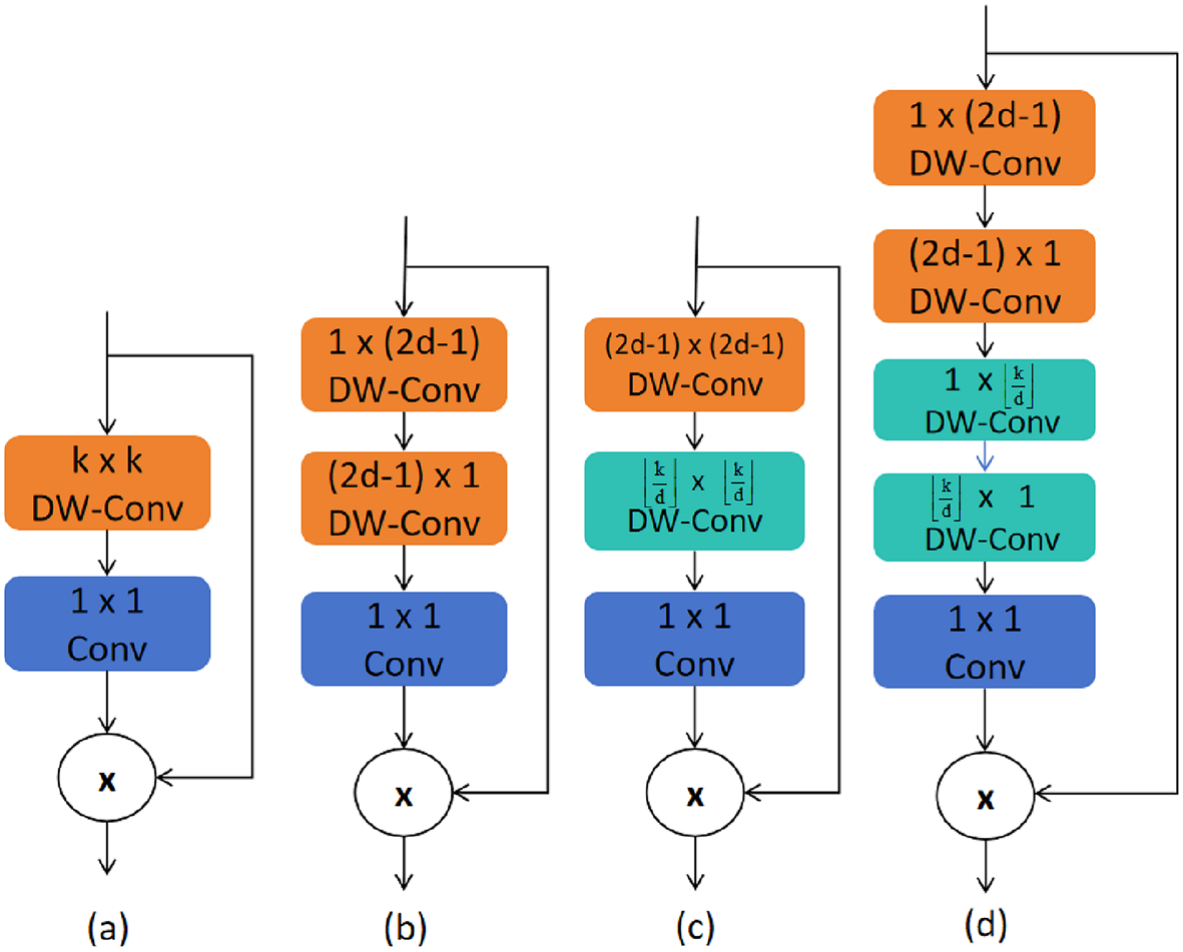
Structure of LSKA.

**Fig. 4:**
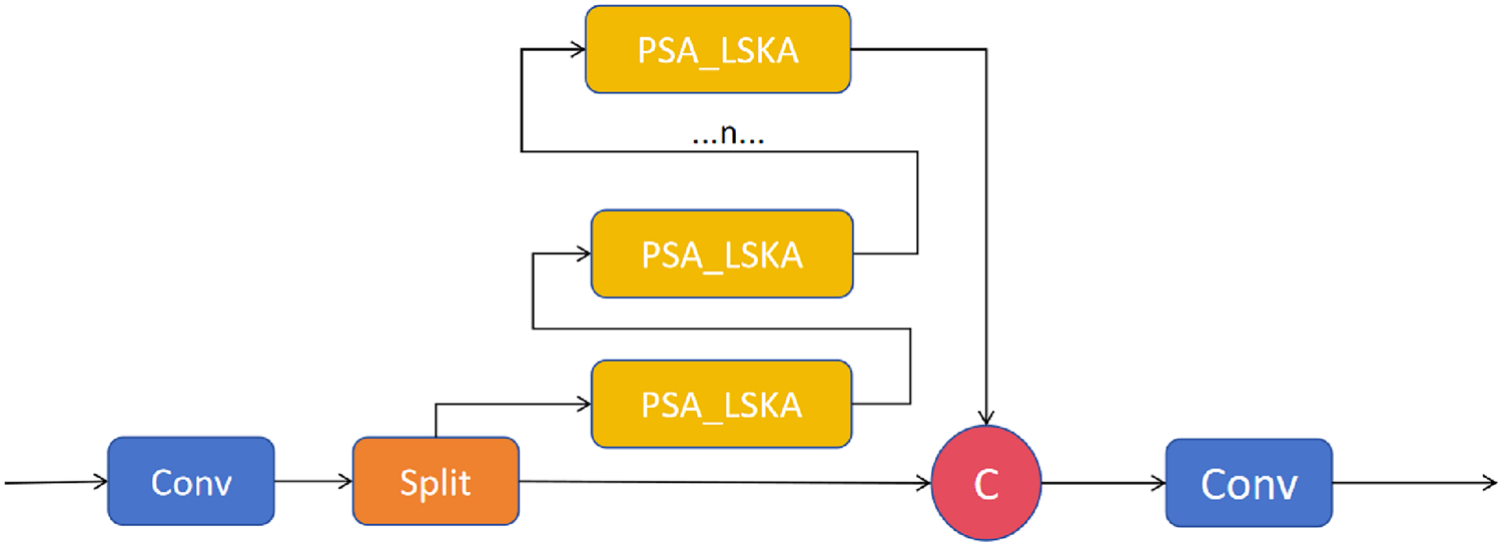
Structure of CLSKA.

In tasks involving traffic sign detection, the vast scale range and complex background interference have always been the core bottlenecks limiting the robustness of detectors. This paper integrates the Large Separable Kernel Attention (LSKA) module^33^, which reduces complexity by decomposing a two-dimensional large kernel into cascaded horizontal-vertical one-dimensional(1D) convolutions, thereby assigning dynamic large-scale weights to each pixel with minimal additional computational/memory overhead. It couples large-scale separable convolutions with channel attention, encoding global context into per-pixel weights while maintaining linear complexity, thereby achieving joint optimization of “where to look” (spatial attention) and “what to look at” (channel attention). LSKA and its variants are shown in Fig. 3a–d are, in order, LKA-trivial, LSKA-trivial, LKA, and LSKA. Based on the architecture shown in Fig. 3, we integrated LSKA into the parallel branches of C2PSA, as illustrated in Fig. 4: Each branch incorporates LSKA while retaining the original PSA self-attention mechanism, thereby forming the “CLSKA” module. This design enables each pixel to simultaneously acquire global self-attention relationships and capture a broader local context through LSKA, achieving a local-global receptive-field enlargement with only a negligible increase in parameters (a few 1$\times$1 projection weights).

LSKA is based on the LKA block design and does not use extended depth convolutions, as shown in Fig. 3a. The input feature map $F \in \mathbb {R}^{C \times H \times W}$, where C is the number of input channels, and H and W represent the height and width of the feature map, respectively. A simple way to design LKA is to use a large convolution kernel in two-dimensional(2D) depth convolutions, i.e., LKA-trivial, with the calculation formula shown in (2)(3)(4):

2 $$\begin{aligned} Z^{C} = \sum _{H, W} W_{k \times k}^{C} * F^{C} \end{aligned}$$

3 $$\begin{aligned} A^{C} = W_{1 \times 1} * Z^{C} \end{aligned}$$

4 $$\begin{aligned} \bar{F}^{C} = A^{C} \otimes F^{C} \end{aligned}$$

The $Z^{C}$ is the deep convolutional output, the $A^{C}$is attention map, and the $\otimes$ is Hadamard product. It can be seen that the computational cost of deep convolutions in this structure increases quadratically with the size of the kernel. To mitigate the high computational cost of deep convolutions with large kernel sizes in LKA-trivial, the authors in^42^ decomposed the original LKA module’s large-kernel deep convolutions into small-kernel deep convolutions and expanded large-kernel deep convolutions, followed by expanded deep convolutions with significantly larger kernels (Fig. 3c). This type of large-kernel decomposition helps alleviate the quadratic increase in computational cost caused solely by deep convolutions and large kernel sizes. As described in^42^, the formulas for LKA are (5), (6), (7), and (8). Among them, *d* is the dilation rate, and $\bar{Z^{C}}$ denotes the output of the depthwise convolution with a kernel size of $(2d-1) \times (2d-1)$. This output can capture local spatial information and compensate for the gridding effect caused by the subsequent depthwise convolution; the kernel size of the subsequent depthwise convolution is $\left\lfloor \frac{k}{d}\right\rfloor \times \left\lfloor \frac{k}{d}\right\rfloor$ (where $\left\lfloor \cdot \right\rfloor$ represents the floor function), while the dilated depthwise convolution is responsible for capturing the global spatial information of the depthwise convolution output ( $\bar{Z^{C}}$ ).

5 $$\begin{aligned} \bar{Z^{C}} = \sum _{H, W} W_{(2 d-1) \times (2 d-1)}^{C} * F^{C} \end{aligned}$$

6 $$\begin{aligned} Z^{C} = \sum _{H, W} W_{\left[ \frac{k}{d}\right] \times \left[ \frac{k}{d}\right] }^{C} * \bar{Z^{C}} \end{aligned}$$

7 $$\begin{aligned} A^{C} = W_{1 \times 1} * Z^{C} \end{aligned}$$

8 $$\begin{aligned} \bar{F}^{C} = A^{C} \otimes F^{C} \end{aligned}$$

We decompose the original two-dimensional(2D) kernels of the depth-wise and dilated depth-wise layers into two consecutive one-dimensional(1D) separable kernels; this four-layer re-arrangement converts LKA into LSKA (Fig. 3d). The same decomposition can be applied to the trivial variant, yielding LSKA-trivial (Fig. 3b). The output calculation of LSKA is given by formulas (9), (10), (11), and (12).

9 $$\begin{aligned} \bar{Z^{C}} = \sum _{H, W} W_{(2 d-1) \times 1}^{C} * \left( \sum _{H, W} W_{1 \times (2 d-1)}^{C} * F^{C}\right) \end{aligned}$$

10 $$\begin{aligned} Z^{C} = \sum _{H, W} W_{\left[ \frac{k}{d}\right] \times 1}^{C} * \left( \sum _{H, W} W_{1 \times \left[ \frac{k}{d}\right] }^{C} * Z^{C}\right) \end{aligned}$$

11 $$\begin{aligned} A^{C} = W_{1 \times 1} * Z^{C} \end{aligned}$$

12 $$\begin{aligned} \bar{F}^{C} = A^{C} \otimes F^{C} \end{aligned}$$

### A dynamic receptive field module

NAM^38^ adopts the module integration approach from CBAM^43^ and redesigns the channel and spatial attention submodules. In the channel attention submodule, the scaling factor of Batch Normalization (BN)^44^ is used to measure the variance of the channel and represent its importance. We designed NAST based on NAM, which uses a hybrid gating mechanism combining Sigmoid and Tanh to enhance more granular control over the distribution of attention weights. BN is shown in Formula (13).

**Fig. 5:**
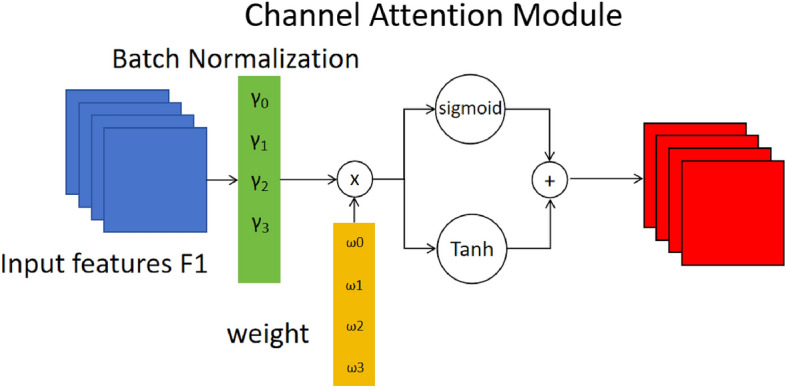
Structure of channel attention module of NAST.

13 $$\begin{aligned} B_{\textrm{out}} = BN(B_{\textrm{in}}) = \gamma \frac{B_{\textrm{in}} - \mu _{B}}{\sqrt{\sigma _{B}^{2} + \epsilon }} + \beta \end{aligned}$$

Among them, $\mu _B$ and $\sigma _B$ are the mean and standard deviation of small batch *B*, respectively; $\gamma$ and $\beta$ are trainable affine transformation parameters (scale and offset)^44^, $\gamma$ is the learnable channel-wise scale vector of BatchNorm, whose elements $\gamma _{i}$ quantify channel variance. The output feature $M_c$ of the channel attention submodule is expressed as:

14 $$\begin{aligned} M_{c} = \textrm{sigmoid}\left( W_{\gamma }\left( BN\left( F_{1}\right) \right) \right) + \tanh \left( W_{\gamma }\left( BN\left( F_{1}\right) \right) \right) \end{aligned}$$

Where W$\gamma$ is the L1-normalized weight vector which can be obtained through $W_{\gamma } = \gamma _{i} / \sum _{j=0} \gamma _{j}$ , the channel attention submodule shown in Fig. 5. No extra parameters are introduced-only the existing BN coefficients are reused for lightweight attention.

In the spatial dimension, the BN scaling factor is also applied to measure the importance of pixels, referred to as pixel normalization. The corresponding output Ms of the spatial attention submodule can be expressed as in formula (15).

15 $$\begin{aligned} M_{s} = \textrm{sigmoid}\left( W_{\lambda }\left( BN_{s}\left( F_{2}\right) \right) \right) + \tanh \left( W_{\lambda }\left( BN_{s}\left( F_{2}\right) \right) \right) \end{aligned}$$

**Fig. 6:**
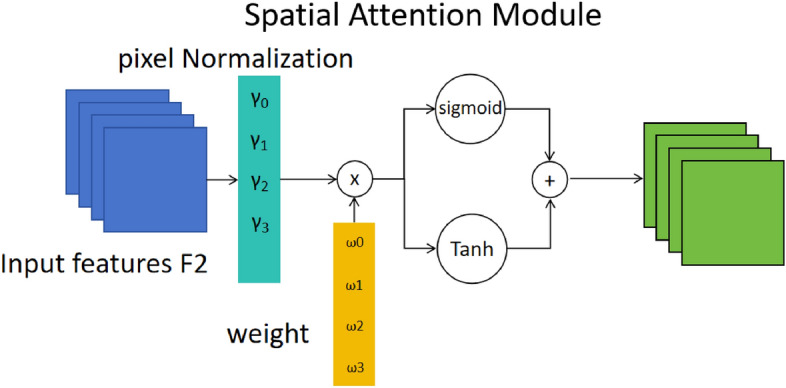
Structure of spatial attention module of NAST.

Where $\gamma$ is the scaling factor, and the weight W$\gamma$ is obtained by $W_{\lambda } = \lambda {i} / \sum _{j=0} \lambda {j}$. The spatial attention submodule is shown in the Fig. 6.

In order to suppress insignificant weights, a regularization term was added to the loss function, with the specific formula as follows:

16 $$\begin{aligned} \textrm{Loss} = \sum _{(x, y)} l(f(x, W), y) + p \sum _{\gamma } g(\gamma ) + p \sum _{\lambda } g(\lambda ) \end{aligned}$$

Where *x* represents the input, *y* represents the output, *W* represents the network weights, $l(\cdot )$ represents the loss function; $g(\cdot )$ represents the 1-norm penalty function; *p* represents the penalty balancing $g(\gamma )$ and $g(\lambda )$.

### Improved loss function

In the YOLOv11 baseline model, the CIoU (Complete-IoU) loss function is used for bounding box regression tasks. Although CIoU performs well in traditional scenarios, it exhibits the following critical flaws in the small object detection scenario of traffic signs: CIoU optimizes bounding box shape alignment by introducing an aspect ratio penalty term, but its definition of aspect ratio lacks a clear geometric interpretation, making it difficult to accurately reflect the true impact of traffic sign width-height differences on model confidence. To overcome the above limitations, we introduce the Normalized Wasserstein Distance (NWD) loss function^37^. The core idea is to model the bounding box of traffic signs as a two-dimensional Gaussian distribution and measure the similarity of the distribution between the predicted box and the real box using optimal transport theory (Wasserstein distance).

In NWD, the boundary box is first modeled as a two-dimensional (2D) Gaussian distribution. For two 2D Gaussian distributions, $\mu _{1} = \mathcal {N}\left( \textbf{m}_{1}, \boldsymbol{\Sigma }_{1}\right)$ and $\mu _{2} = \mathcal {N}\left( \textbf{m}_{2}, \boldsymbol{\Sigma }_{2}\right)$, the second-order Wasserstein distance can be defined as:

17 $$\begin{aligned} W_{2}^{2}\left( \mu _{1}, \mu _{2}\right) = \left\| \textbf{m}_{1} - \textbf{m}_{2}\right\| _{2}^{2} + \operatorname {Tr}\left( \boldsymbol{\Sigma }_{1} + \boldsymbol{\Sigma }_{2} - 2\left( \boldsymbol{\Sigma }_{2}^{1 / 2} \boldsymbol{\Sigma }_{1} \boldsymbol{\Sigma }_{2}^{1 / 2}\right) ^{1 / 2}\right) \end{aligned}$$

The formula can also be simplified to:

18 $$\begin{aligned} W_{2}^{2} \left( \mu _{1}, \mu _{2} \right) = \left\| {m}_{1}-{m}_{2} \right\| _{2}^{2}+ \left\| { \sum }_{1}^{1/2}-{ \sum }_{2}^{1/2} \right\| _{F}^{2} \end{aligned}$$

where ${\left| \left| \cdot \right| \right| }_{F}$ is the Frobenius norm.

For boundary boxes $A=\left( c x_{a}, c y_{a}, w_{a}, h_{a}\right) \text{ and } B=\left( c x_{b}, c y_{b}, w_{b}, h_{b}\right)$ modeled Gaussian distributions Na and Nb, this can be further simplified to:

19 $$\begin{aligned} W_{2}^{2}\left( \mathcal {N}_{a}, \mathcal {N}_{b}\right) = \left\| \left[ \left[ c x_{a}, c y_{a}, \frac{w_{a}}{2}, \frac{h_{a}}{2}\right] ^{\textrm{T}}, \left[ c x_{b}, c y_{b}, \frac{w_{b}}{2}, \frac{h_{b}}{2}\right] ^{\textrm{T}}\right] \right\| _{2}^{2} \end{aligned}$$

$W_{2}^{2}\left( \mathcal {N}_{a}, \mathcal {N}_{b}\right)$is a distance measure and cannot be directly used as a similarity measure (i.e., values between 0 and 1, such as IoU), so it is normalized using its exponential form to obtain a new measure, NWD:

20 $$\begin{aligned} NWD\left( N_{a}, N_{b}\right) = \exp \left( -\frac{\sqrt{W_{2}^{2}\left( N_{a}, N_{b}\right) }}{C}\right) \end{aligned}$$

Where C is a constant closely related to the dataset, empirically set to the average absolute size of AI-TOD’s c in the experiment, achieving optimal performance and robustness within a certain range. Therefore, the NWD loss function is designed as:

21 $$\begin{aligned} \mathcal {L}_{NWD} = 1 - NWD\left( N_{p}, N_{g}\right) \end{aligned}$$

Where Np is the Gaussian distribution model of the predicted box P, and Ng is the Gaussian distribution model of the true bounding box G.

## Experiments

### Datasets

**Fig. 7:**
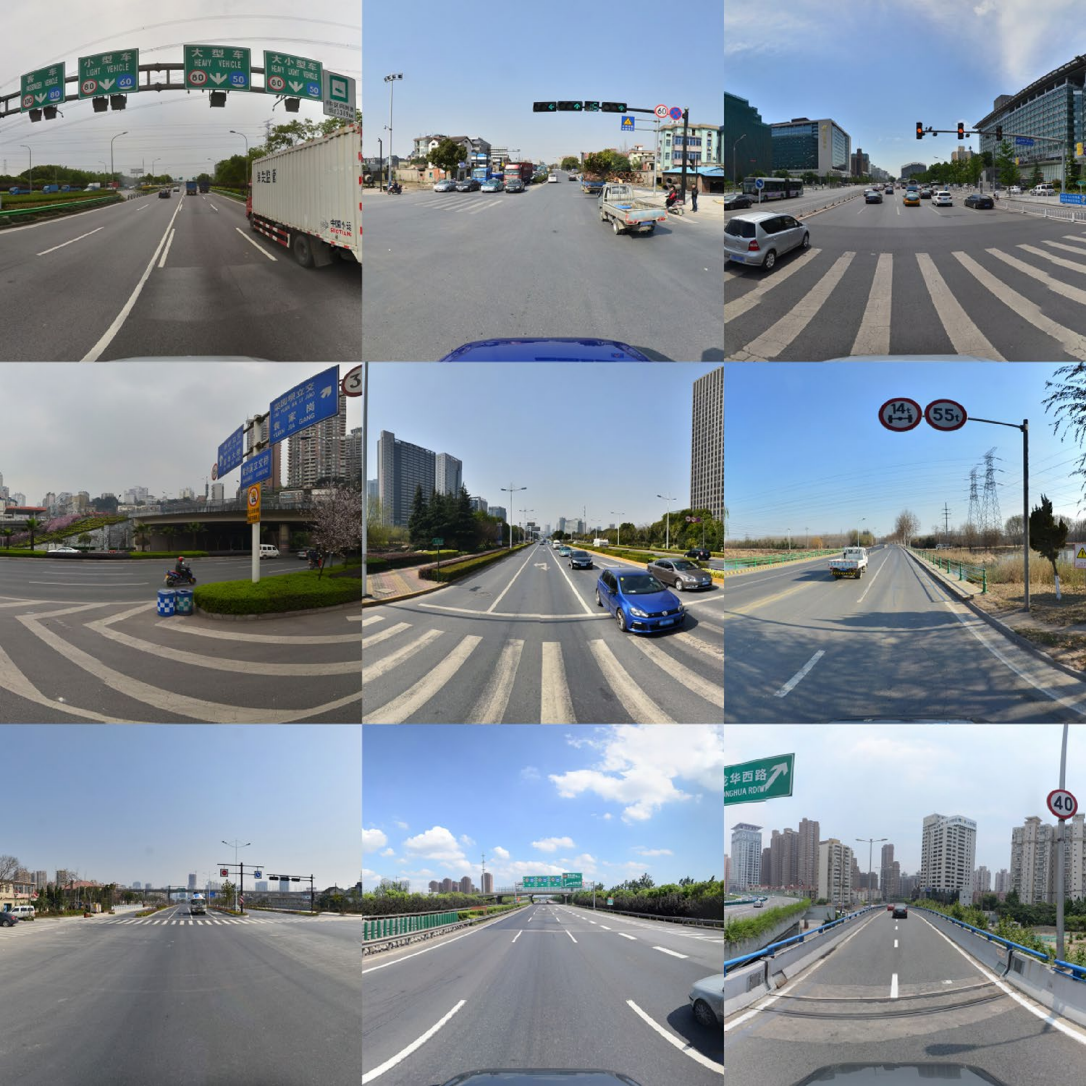
Sample images from the TT100K public benchmark dataset^1^. Reproduced with permission from the copyright holders under a CC BY open access license.

This study selected the TT100K (Tsinghua-Tencent 100K) public benchmark dataset to construct an experimental environment for systematically evaluating the robustness of traffic sign detection models under multi-scenario and cross-regional conditions. TT100K is designed for Chinese urban roads and features a large scale, diverse categories, and complex environments. Tsinghua University and Tencent Street View Lab jointly developed the TT100K dataset. Tencent’s Street View fleet captured the original images during operations on main and secondary roads in over 30 Chinese cities, as shown in Fig. 7.

This paper employs a filtering strategy that retains only the 45 categories that occur at least 100 times and reclassifies them in a 7:2:1 ratio. This yields a training set of 6, 793 images, a test set of 1, 949 images, and a validation set of 996 images, for a total of 9, 738 images used for model training and performance evaluation.

The GTSDB dataset, jointly released by Darmstadt University of Technology and Hannover University in 2013, stands as one of Europe’s earliest publicly available traffic sign detection benchmarks. Selected data is illustrated in Fig. 2. Its raw data was captured using an industrial camera mounted behind a vehicle windshield along German Autobahn and urban ring road sections, comprising 900 PPM images at 1360$\times$800 resolution. The dataset features official annotations for 43 fine-grained sign categories. Considering the redundancy of fine-grained categories in practical deployment, this paper maps them to four major categories-”Prohibitory, Mandatory, Warning, and Other”-based on the official category tree. After uniformly converting the images to JPEG format, the dataset was randomly split into an 8:2 ratio: 720 images for the training set and 180 images for the test set. This configuration enables cross-domain evaluation against the TT100K dataset to validate the model’s generalization capabilities across different national traffic sign styles and scene conditions.

### Experiment environment

Our detailed experimental environment configuration is shown in Table 1. This study was conducted on a heterogeneous computing node with four NVIDIA Quadro RTX 6000 graphics cards, each with 24 GB of GDDR6 memory. The node has dual Intel Xeon Gold 5218 CPUs with a clock speed of 2.30 GHz and 20 cores/40 threads, as well as 187 GB of DDR4 ECC memory. The operating system used is Ubuntu 20.04.6 LTS (kernel 5.15), and the programming language is Python 3.10.18. We selected the PyTorch 2.7.1 deep learning framework, combined with CUDA 12.6 and cuDNN 8.9.5.

**Table 1:** Experimental configuration environment information.

Hardware and Software configuration	Parameters
Operating System	Ubuntu 20.04.6 LTS
CPU	Intel(R) Xeon(R) Gold 5218
GPU	NVIDIA QUADRO RTX 6000
Operational Memory	187GB
Graphics Memory	24GB
Python Version	3.10.18
Pytorch Version	2.7.1
CUDA Version	12.6

The hyperparameters for training can be seen in Table 2. Our input images are uniformly scaled to $640\times 640$ pixels, and random horizontal flipping, Mosaic, and MixUp data augmentation are used to improve the model’s generalization performance. The optimizer selected is SGD with momentum (momentum=0.937, weight decay=$5\times 10^{-4}$). The initial learning rate and final learning rate are both set to 0.01, and dynamic adjustment is performed using a cosine annealing strategy. The batch size is set to 128. The number of training epochs for the TT100K dataset is set to 300 epochs.

**Table 2:** Key parameter values of network training.

Key parameters	Parameter value
Epochs	300
Image size	$640\times 640$
Batch	128
Optimizer	SGD
Initial learning rate	0.01
Momentum	0.937
Weight decay	0.0005
Patience	50
Workers	8
lrf	0.01
lr0	0.01
Close_mosaic	10

### Evaluation metrics

#### Accuracy of the detection indicator

Precision measures the proportion of true positive samples among the results predicted by the model as positive samples, directly reflecting the model’s ability to suppress false positives. Precision P represents the proportion of correctly identified positive samples across all samples, and higher detection precision indicates a higher probability of the model correctly identifying the object. Formula (22) illustrates the calculation method for precision P. In autonomous driving applications, high precision effectively prevents erroneous braking or steering caused by false positives, thereby enhancing system safety. Recall rate describes the proportion of all true traffic signs that are successfully detected, and Formula (23) shows the calculation formula for recall rate.

22 $$\begin{aligned} Precision = TP / (TP + FP) \end{aligned}$$

23 $$\begin{aligned} Recall = TP / (TP + FN) \end{aligned}$$

In the field of traffic sign detection, average precision (AP) is a key indicator for evaluating model performance. Its calculation involves integrating the precision-recall (PR) curves for each traffic sign category at different intersection over union (IoU) thresholds. Specifically, the AP value is calculated using the following formula:

24 $$\begin{aligned} AP=\int _{0}^{1}P(r)dr \end{aligned}$$

Among them, P(r) represents the PR curve function at a specific IoU threshold t. In addition, the mean average precision (mAP) is obtained by averaging the AP values of all categories, and its calculation formula is:

25 $$\begin{aligned} {mAP}_{t}=\frac{1}{N}\displaystyle \sum _{i=1}^{N}{AP}_{t, i} \end{aligned}$$

#### Model scale and computational metrics

When evaluating deep learning models, particularly those deployed on automotive electronic control units (ECUs) or mobile device system-level SoCs, three core performance metrics are widely used: parameters, floating-point operations per second (FLOPs), and frames per second (FPS). Three numbers define a vehicular model: Params (M) for storage, FLOPs (G) for power, and $640\times 640$ FPS for real-time.

### Comparison experiments

#### Qualitative comparative analysis

**Fig. 8:**
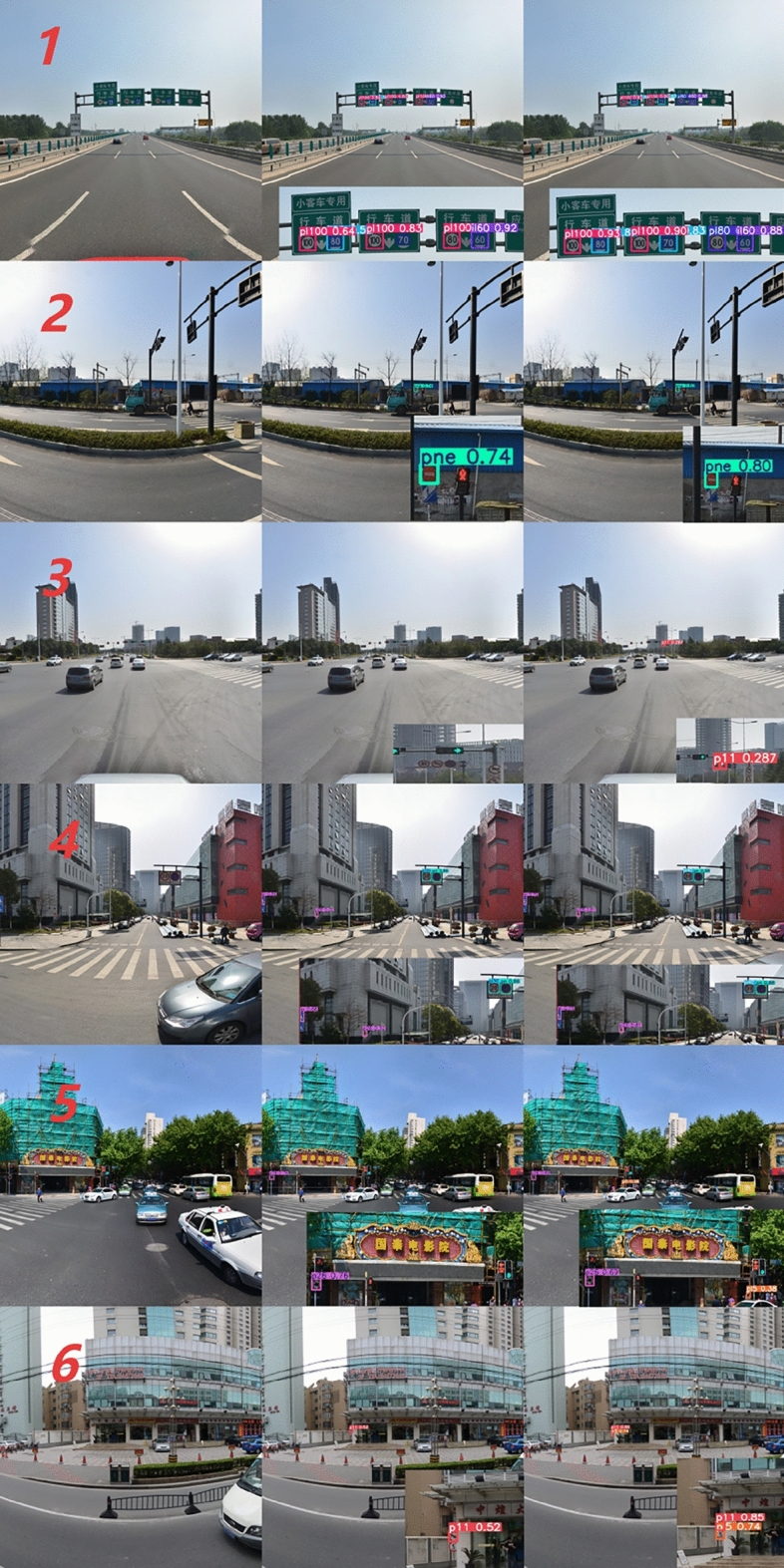
Detection results comparison between YOLOv11s (middle) and YOLO-AML (right column) on TT100K dataset sample images. Images reproduced with permission under a CC BY open access license. Bounding boxes and confidence scores are model predictions.

Figure 8 shows a comparative analysis of the detection performance between the improved traffic sign detection model and the YOLOv11s model. The selected images cover various traffic scenarios, including high-density multi-object, single small objects, objects with occlusions, distant small objects, and complex environments with multi-scale objects. From the detection results, it can be seen that the YOLOv11s model has certain limitations when detecting multi-scale traffic signs in complex scenes, while the improved model demonstrates significantly superior detection performance compared to YOLOv11s.

This study presents detection results under six different conditions. The first group demonstrates performance in detecting small and densely packed traffic signs, while the second group shows performance in detecting small single traffic signs. In the first group, YOLOv11s had missed detections, whereas the improved model detected all traffic signs. In the second group, the improved model also achieved higher detection confidence. From the detection results of the first two groups, it can be observed that the improved model achieves outstanding performance in detecting small objects, indicating a significant improvement in detection performance. For traffic signs of the same category, the improved model consistently demonstrates higher detection confidence compared to YOLOv11s. This is primarily attributed to the improved model retaining more fine-grained information through the SPD-Conv module, making the features of small objects more prominent. The third, fifth, and sixth groups show the detection results for distant small traffic signs.

As shown in the Fig. 8, the improved model achieves better detection performance for occluded and multi-scale traffic signs. Based on the above detection results, it is evident that the improved model accurately detects multi-scale small objects in various scenarios, with a significant reduction in missed detections. Compared to the original model, the detection performance has been significantly enhanced.

#### Quantitative comparative analysis

**Fig. 9:**
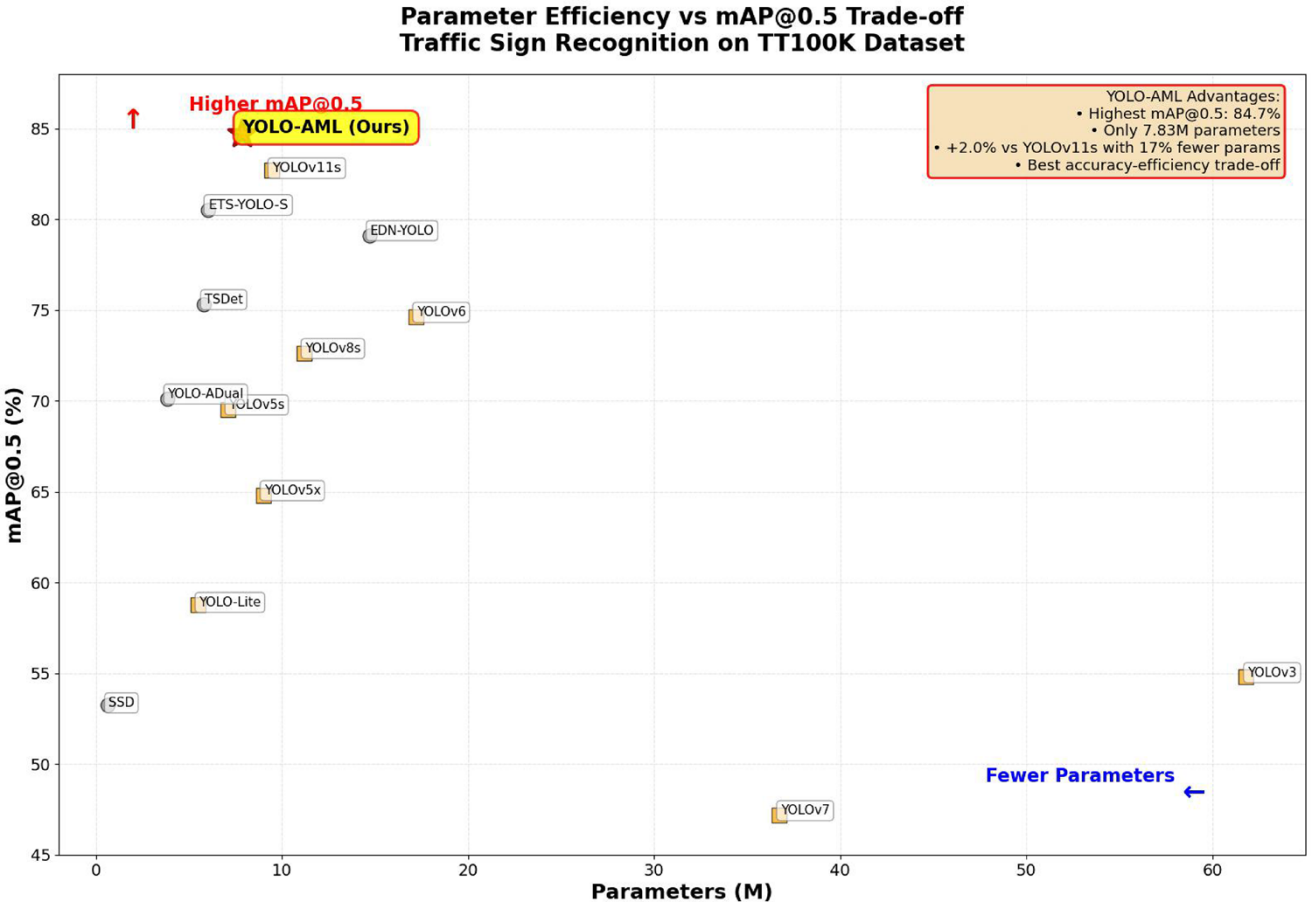
Diagram of Performance efficiency and map@0.5 trade-off on TT100K Dataset. (The yellow square in the diagram represents the original version of YOLO, while the gray circle denotes variants of YOLO or other types of algorithms.).

Table 3 shows the performance metrics of the improved model on the TT100K dataset, where our model achieves an FPS of 178.6 and an mAP of 84.7%, both surpassing existing models(Some of the FPS entries in Table 3 are taken from the referenced studies. ). Figure 9 further visually demonstrates the pronounced advantage of YOLO-AML in achieving the optimal trade-off between mAP@0.5 and parameter efficiency. Notably, compared to the original model, the improved model reduces the number of parameters by approximately 17% and computational complexity by approximately 16.8%, contributing to model lightweighting and enhanced computational efficiency. The detection speed has improved by 72.2 FPS, and the accuracy(mAP@0.5) has increased by 2.0%, significantly enhancing real-time performance and detection accuracy. The improved algorithm demonstrates significant improvements in multi-scale object detection, particularly for objects at short distances, showcasing excellent multi-scale object detection performance.

**Table 3:** Comparison of different models for traffic sign recognition on the TT100K dataset.

Model	Params(M)	GFLOPS	FPS	mAP@0.5
Faster R-CNN^26^(2015)	–	–	40.0	53.3
SSD^23^(2016)	**0.64**	**3.1**	–	53.26
YOLOv3(2018)	61.8	155.5	57.8	54.8
YOLOv5s(2020)	7.1	16.2	156.3	69.5
YOLOX(2021)	9.0	26.7	66.5	64.8
YOLO-Lite(2018)	5.5	16.0	112.4	58.8
YOLOv6(2022)	17.2	44.1	148.8	74.6
YOLOv7(2022)	36.7	103.9	109.9	47.2
YOLOv8s(2023)	11.2	28.7	–	72.6
YOLOv10(2024)	–	24.6	83	82.4
EDN-YOLO^5^(2024)	14.7	26.0	131.6	79.1
TSDet^6^(2024)	5.8	46.8	82	75.3
ETS-YOLO-S^36^(2025)	6.0	14.3	–	80.5
YOLO-ADual^3^(2024)	3.82	11.2	–	70.1
YOLOv12s(2025)	9.25	21.3	72.2	40.4
YOLOv13s(2025)	9.02	20.8	53.5	10.7
RT-DETR(2023)	32.09	104.0	71.9	24.5
YOLOv11s(2024)	9.43	21.4	106.4	82.7
Ours	7.83	17.8	**178.6**	**84.7**

Bold values denote optimal results, defined as lower values for parameters and computational complexity, or higher values for accuracy and speed.

Compared to other mainstream models, the computational complexity of the improved model is 17.8 GFLOPS, while that of YOLOv5s is 16.2 GFLOPS. In comparison, the computational complexity of the improved model is slightly higher, exceeding YOLOv5s by 1.6 GFLOPS, representing an increase of approximately 9.9%. Although the computational complexity of the improved model is slightly higher, its accuracy (mAP@0.5) reaches 84.7, which is 15.2% higher than YOLOv5s’s 69.5.

**Table 4:** Comparison of the model effects for the original improved models on the GTSDB dataset.

Categories	YOLOv11s		Ours	
	mAP50(%)	mAP50:95(%)	mAP50(%)	mAP50:95(%)
Prohibitory	98.9	83.4	99.2	84.4
Mandatory	88.2	71.0	90.4	73.2
Danger	96.7	79.9	96.5	79.4
Other	93.6	76.2	95.5	78.8
All	**94.4**	77.6	**95.2**	78.9

Bold values denote optimal results, defined as lower values for parameters and computational complexity, or higher values for accuracy and speed.

From the perspective of parameter count, the improved model has 7.83 million parameters, which meets the requirements for deployment on autonomous driving embedded devices while maintaining high performance with a relatively low parameter count. In terms of accuracy (mAP@0.5), the improved model achieves an accuracy of 84.7%, the highest among all listed models. Compared to EST-YOLO-S, accuracy has improved by 4.2%. Compared to YOLOv12s, YOLOv13s, and RT-DETR, YOLO-AML contains only 7.83 million parameters and 17.8 GFLOPs-reductions of 21%, 15%, and 83%, respectively-yet achieves 84.7% on TT100K mAP@0.5 on the TT100K dataset, outperforming the aforementioned models by 44.3%, 74.0%, and 60.2%, respectively. This performance is driven by SPD-Conv’s lossless downsampling preserving fine texture details, CLSKA’s separable large kernels expanding the receptive field without increasing parameters, NAST’s reuse of BN scaling factors to suppress complex backgrounds, and NWD’s provision of stable gradients for targets. Consequently, the FPS is boosted to 178.6, fully demonstrating its comprehensive advantages in real-time small traffic sign perception.

In terms of detection speed, the improved model achieved a detection speed of 178.6 FPS, compared to the YOLOv5s model (detection speed of 156.3 FPS). Despite the fact that our model increased in parameters and computational complexity by 0.73M and 1.8 GFLOPS, respectively, our model’s detection speed has still improved by 22.3 FPS. Compared to the EDN-YOLO model (131.6 FPS), our model’s detection speed has improved by 47 FPS. However, the EDN-YOLO model has significantly higher parameter counts and computational requirements than our model. This indicates that our model achieves performance improvements while reducing parameter counts and computational requirements, giving it a clear advantage in real-time performance and enabling faster processing of input data. Overall, our model outperforms other models in terms of accuracy and detection speed, while also achieving a good balance in terms of parameter count and computational complexity. Compared to mainstream models, our model demonstrates significant improvements in detection accuracy across different scales, indicating that it possesses better adaptability and robustness when handling multi-scale objects.

To evaluate YOLO-AML’s generalization capabilities across different regions and scenarios, we conducted additional experiments on the GTSDB (German Traffic Sign Detection Benchmark) dataset. GTSDB features urban road scenes in Germany, exhibiting significant differences in image style, lighting conditions, and occlusion types compared to TT100K. This dataset effectively validates the model’s adaptability to European traffic sign styles and complex environmental distractions. As shown in Table 4, YOLO-AML achieves a 95.2%mAP@0.5 on GTSDB, representing a 0.8% improvement over YOLOv11s. Similar gains are observed compared to the TT100K dataset, indicating stable accuracy improvements across datasets.

Specifically, for prohibitory signs-which frequently appear in complex urban settings within GTSDB and are highly susceptible to background noise interference-YOLO-AML demonstrated the most significant average accuracy improvement over the baseline. This is directly attributable to the powerful, parameter-free attention mechanism provided by the NAST module. NAST successfully suppressed interference from complex road environment backgrounds. YOLO-AML demonstrates the most significant average accuracy improvement over the baseline for these signs, directly attributable to the powerful, parameter-free attention mechanism provided by the NAST module. NAST successfully suppresses complex road environment backgrounds, ensuring the purity of these critical sign features. For other sign categories, where size and distance vary significantly across different scenes, YOLO-AML also achieves robust accuracy gains. YOLO-AML maintains high performance on GTSDB primarily due to its structurally versatile design. The CLSKA module’s adaptive multi-scale feature aggregation capability enables it to effectively handle objects of varying sizes within GTSDB-from the minute targets in TT100K to the larger signs in GTSDB. Concurrently, the normalization-based parameter-free attention mechanism in the NAST module demonstrates robustness when processing novel backgrounds and noise distributions.

The application of the NWD loss function across all categories ensures precise measurement of minor localization errors, safeguarding the model’s robust regression performance. Through precise feature channel adjustment, NAST effectively suppresses background interference across diverse image domains, ensuring feature purity and thereby enhancing the model’s generalization accuracy. In summary, YOLO-AML maintains lightweight architecture, high accuracy, and strong anti-interference characteristics across different scenarios and non-TT100K distributions. The synergistic effects of its innovative modules enable cross-regional generalization capabilities.

### Ablation study

From Table 5 in the ablation experiment, we can see that by analyzing the performance improvement of each innovation point on YOLOv11s module by module, the four improvements achieve cascading gains in small object detection performance while maintaining computational efficiency.

First, the YOLOv11s baseline achieves only 0.827 mAP@0.5 under conditions of 9.43M parameters and 21.4GFLOPs, exposing the irreversible compression of fine-grained features of small objects by traditional stride convolution and pooling. Experimental results show that after replacement, the parameters are reduced to 7.96M, the computation is reduced to 17.9GFLOPs, and the precision is increased from 0.828 to 0.841, verifying the effectiveness of SPD-Conv in maintaining small object textures while being lightweight.

NAST utilizes the scaling factor $\gamma$ of BatchNorm as attention weights, constructing zero-parameter channel-space joint suppression between the detection head and backbone terminal. Channels with $\gamma$ close to 0 are treated as background noise, and their features are adaptively suppressed, thereby improving the signal-to-noise ratio without increasing any convolution overhead. Experiments show that after adding NAST, Recall increases from 0.730 to 0.733, mAP@0.5s increase from 0.824 to 0.829, and FPS increases from 166 to 172, indicating that redundant computations are reduced due to background suppression, while inference speed is slightly improved.

CLSKA replaces LKA’s two-dimensional large-kernel convolutions with cascaded hollow depth separable convolutions and C2PSA attention fusion, trading linear parameter growth for quadratic receptive field expansion. Experimental results show that CLSKA’s Recall improved from 0.733 to 0.752, while map@0.5 slightly decreased, and the number of parameters further decreased to 7.83M, with GFLOPs dropping to 17.8, validating its lightweight design that achieves performance gains without sacrificing performance while expanding the receptive field.

Finally, the NWD loss function addresses the issue of gradient vanishing caused by slight offsets resulting in zero IoU in small object localization by modeling bounding boxes as two-dimensional Gaussian distributions and replacing IoU with Wasserstein distance. NWD provides stable gradients even when predicted boxes have no overlap or completely contain the ground truth boxes, significantly improving label assignment and regression accuracy. Experimental results show that after incorporating NWD, the mAP@0.5 improved from 0.824 to 0.847, and Precision improved from 0.843 to 0.865, while parameters and computational complexity remained unchanged, and FPS increased to 178.6. This indicates that NWD significantly enhances the robustness of small object localization with nearly zero inference overhead.

**Table 5:** Comparison of the ablation study results for the improved models on the TT100K dataset.

Model	Precision	Recall	Params(M)	GFLOPS	FPS	mAP@0.5	mAP@0.5-0.95
Yolov11s	0.828	0.734	9.43	21.4	106.4	0.827	0.646
Yolov11s+SPDConv	0.841	0.730	7.96	17.9	166.7	0.824	0.643
Yolov11s+NAST	0.854	0.735	9.44	21.5	179	0.839	0.657
Yolov11s+NWD	0.835	**0.763**	9.44	21.5	**212.8**	0.84	0.659
Yolov11s+CLSKA	0.841	0.730	7.96	17.9	166.7	0.824	0.643
Yolov11s+CLSKA+NAST	0.838	0.727	9.31	21.4	161.3	0.826	0.618
Yolov11s+NAST+NWD	0.853	0.738	9.4	21.5	161.3	0.837	0.641
Yolov11s+CLSKA+NWD	0.856	0.736	9.31	21.4	158.7	0.841	0.648
Yolov11s+SPDConv+NAST	0.859	0.733	7.96	17.9	172.4	0.829	0.645
Yolov11s+SPDConv+NAST+CLSKA	0.843	0.728	**7.83**	**17.8**	169.5	0.824	0.641
YOLO-AML	**0.865**	0.752	**7.83**	**17.8**	178.6	**0.847**	**0.654**

Bold values denote optimal results, defined as lower values for parameters and computational complexity, or higher values for accuracy and speed.

As can be seen from the above analysis, SPD-Conv, NAST, CLSKA, and NWD work synergistically across four dimensions: information fidelity, noise suppression, receptive field expansion, and loss function optimization. Under the lightweight constraints of 7.83 million parameters and 17.8 GFLOPs, they improve the mAP@0.5 from 0.827 to 0.847, achieving a dual breakthrough in small object detection accuracy, computational efficiency, and lightweight design.

Regarding the individual contributions of each module to model performance and their interactions, we first examined the standalone effect of the NAST module. Experimental data show that introducing the NAST module alone on the baseline model significantly improved the mAP@0.5 from 0.827 to 0.839, strongly demonstrating the module’s effectiveness. NAST employs its innovative normalization-based hybrid gating mechanism (sigmoid and tanh) to precisely modulate attention weight distributions without increasing computational overhead. It excels at suppressing complex background noise interference in long-range small object detection scenarios, significantly enhancing feature purity and target recognition capabilities.

Second, the independent ablation results for the CLSKA module are equally encouraging. When embedding only the CLSKA module into the backbone network, we observed another level of substantial performance improvement: accuracy increased from 0.828 to 0.841, parameters decreased from 9.43 to 7.96, and FPS rose from 106.4 to 166.7. Although the mAP@0.5 decreased, the drop was only 0.3%, remaining within an acceptable range. CLSKA combines Lightweight Spatial Kernel Attention (LSKA) with a multi-branch architecture. Its core advantage lies in efficiently capturing and aggregating weak multi-scale feature information while maintaining overall model lightness. For distant small traffic signs with extremely weak feature signals, this efficient multi-scale feature capture capability is crucial for performance assurance.

Next, we analyzed the independent contribution of the NWD loss function. Replacing the traditional IoU loss with NWD in the baseline significantly improved the overallmAP@0.5from 0.824 to 0.84, while recall metrics for small objects also showed a notable increase. By normalizing the Gaussian distribution to measure the overlap of small bounding boxes, the NWD loss function successfully overcomes the gradient vanishing issue inherent in traditional IoU loss during small object regression. This provides the model with more stable and precise localization regression signals.

Most importantly, our experiments clearly demonstrate powerful synergistic effects between modules. When SPDConv, NAST, CLSKA, and NWD are combined to form the complete YOLO-AML network, the model’s performance improvement is not a simple sum of individual contributions but exhibits a synergistic gain exceeding linearity, achieving the optimal performance metrics in this experiment. Specifically, CLSKA delivers rich, high-quality multi-scale features, which NAST subsequently refines through denoising and enhancement, ensuring the detection head receives both clean and discriminative input. NWD then leverages these optimized features at the network’s backend for ultra-precise localization regression. This dual-enhancement mechanism of “high-quality feature extraction” and “precise loss function optimization” fully demonstrates that the four components we propose-SPDConv, NAST, CLSKA, and NWD-are complementary and indispensable within the YOLO-AML architecture. Ultimately, this ensures the model achieves optimal performance in detecting small traffic signs at long distances while meeting lightweight objectives.

**Fig. 10:**
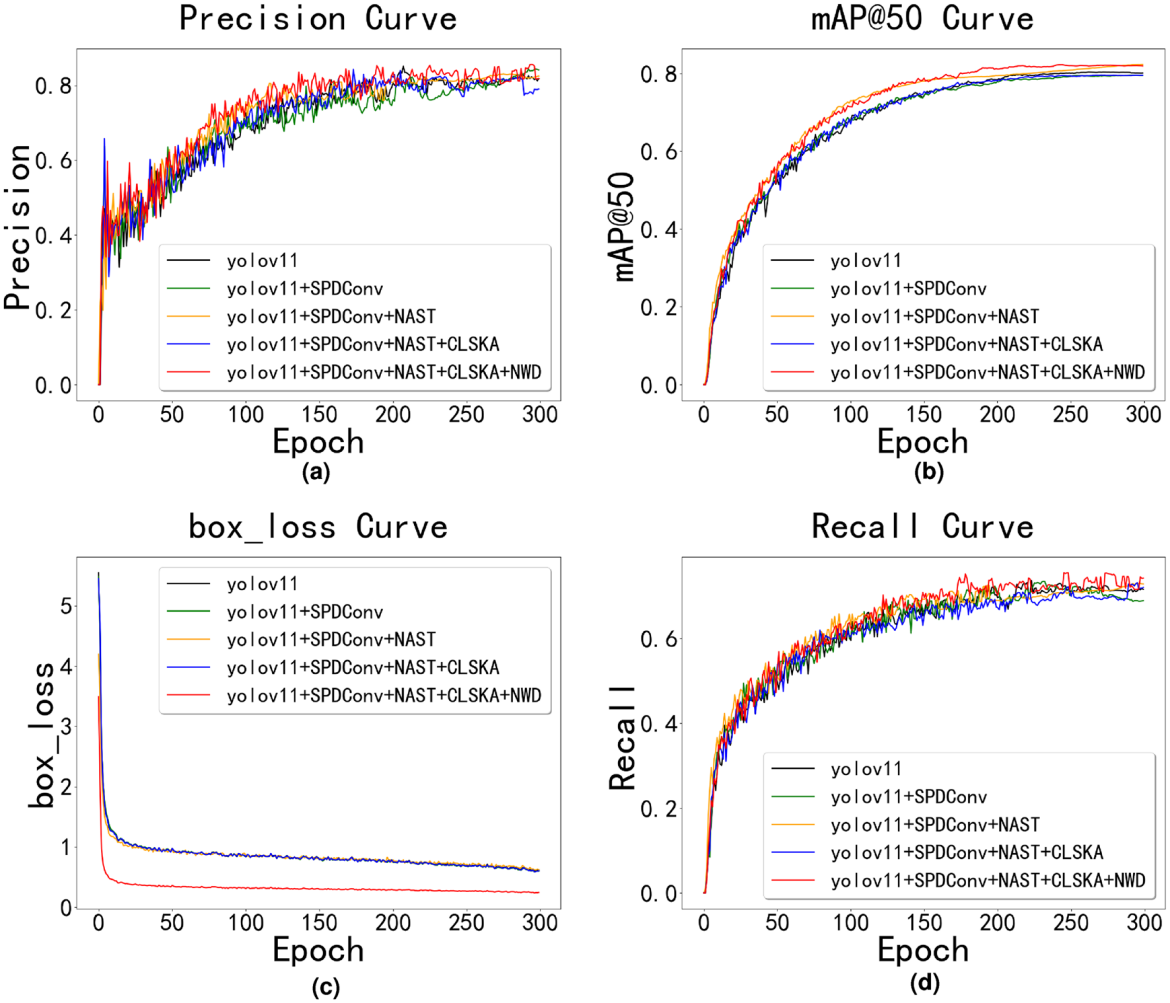
Comparison of training process metrics for different models in ablation experiments.

We also visualized various data metrics during the experimental training process, as shown in Fig. 10. The figure consists of four subplots, which respectively show the changes in model accuracy (Precision) from Fig. 10a, mAP@0.5 (mean Average Precision at IoU=0.5) from Fig. 10b, bounding box loss (box_loss) from Fig. 10c, and recall rate (Recall) from Fig. 10dduring the training process.

### Resource-constrained edge device deployment evaluation

**Fig. 11:**
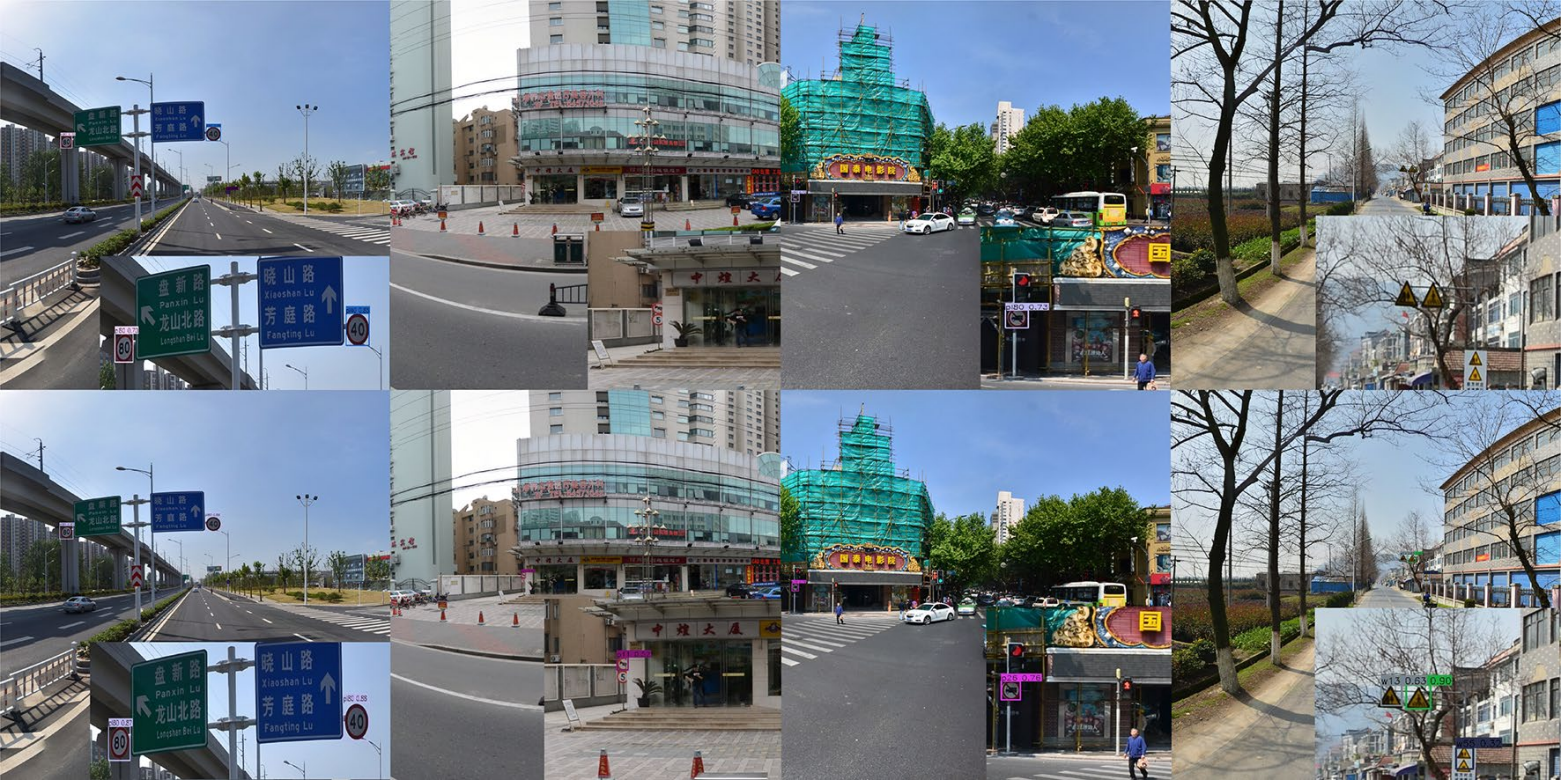
Detection results on Jetson Nano B01 edge device using TT100K dataset sample images. Images reproduced with permission under a CC BY open access license.

We previously validated the effectiveness of YOLO-AML using two public datasets: TT100K and GTSDB. To further assess its suitability for edge deployment, the model was ported to a Jetson Nano B01 with only 4 GB of memory. Testing 12 640$\times$640 images under ONNX Runtime showed that the average inference time of the improved model decreased from 1751 ms to 1515 ms, while the FPS increased from 0.57 to 0.66-a 15% improvement-with a 17% reduction in parameters. As shown in the Fig. 11, the top row displays results from the original model, while the bottom row shows YOLO-AML’s performance. The model accurately captures distant small traffic signs with high confidence even in complex road scenes, fully demonstrating its robustness and efficiency in resource-constrained environments. This validates its potential for real-time, high-accuracy deployment in automotive edge computing scenarios.

### Visualization result

**Fig. 12:**
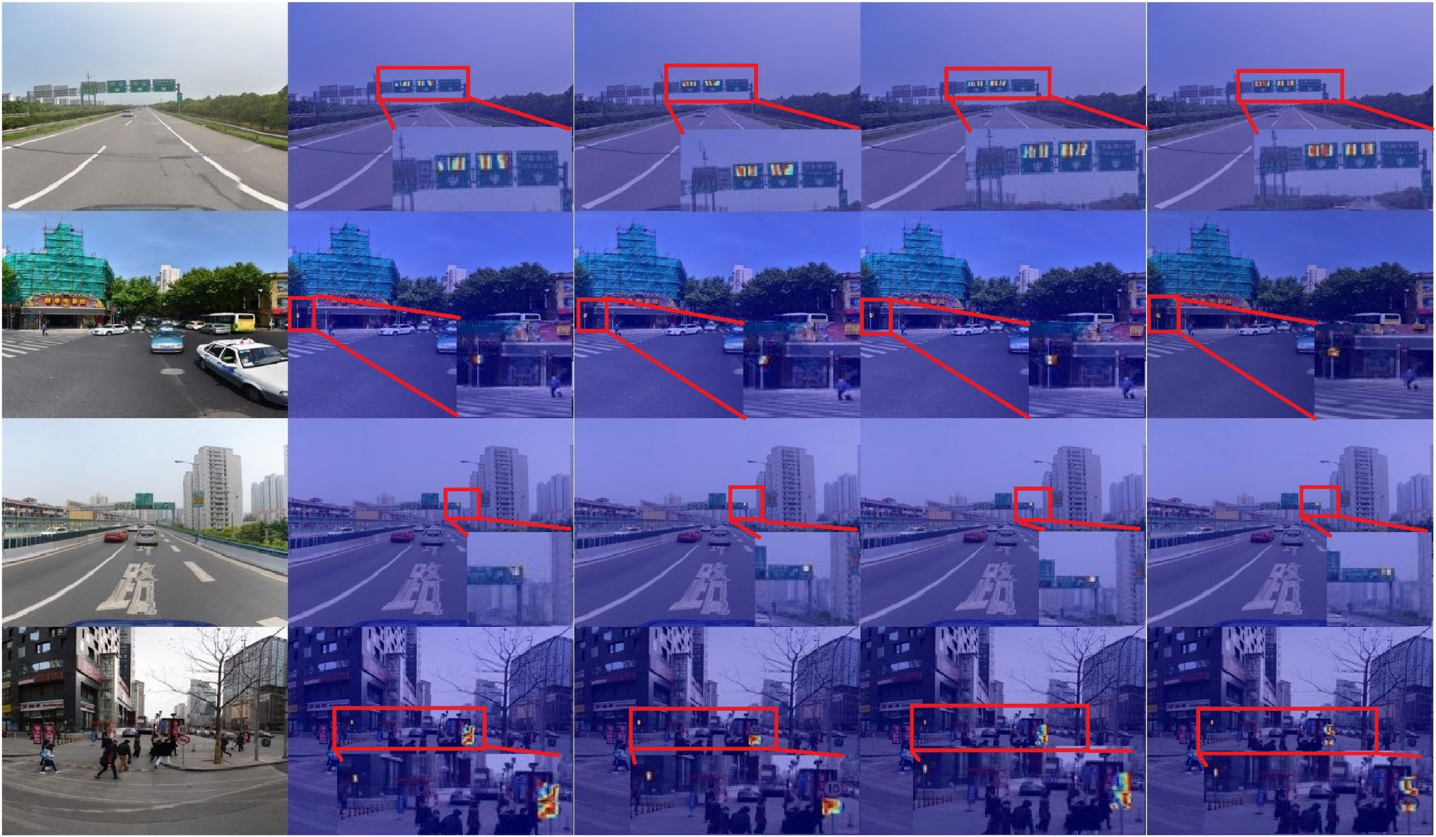
Grad-CAM heatmap visualization comparing feature activation maps across different models on TT100K dataset sample images. Images reproduced with permission under a CC BY open access license. Brighter regions indicate higher activation intensity.

To better visually compare the performance improvements of the original model, we used GradCAM heatmaps for visualization analysis, as shown in Fig. 12. The Grad-CAM visualizations display, from left to right, the original image, the YOLOv11 baseline model, YOLOv8, YOLOv10, and the improved model’s heatmaps. The heatmap sequence from left to right clearly shows varying degrees of diffusion in the activation regions between the original image and the YOLOv11 baseline, YOLOv8, and YOLOv10: while the baseline model can locate signs, the edges of high-response areas are blurred. while simultaneously amplifying background foliage and road texture, indicating loss of fine-grained cues due to shallow subsampling. YOLOv8 and YOLOv10, employing continuous stride convolutions, produce only scattered hotspots for small targets (<0.1% pixel coverage), frequently disrupted by lampposts and shadows, resulting in “hotspot drift. ” In contrast, the YOLO-AML heatmap on the far right displays a bright, compact kernel whose geometric center nearly aligns with the sign’s center. Its edges exhibit sharp contours, while background responses are significantly suppressed.

To demonstrate that the CLSKA module enlarges the receptive field, we apply Grad-CAM to the final feature map of the Neck. As shown in the forth column of Fig. 12, the heatmap produced with CLSKA exhibits larger and more continuous high-response regions than the baseline, indicating that the network captures richer contextual information around the traffic sign with complete geometric coverage. This visualization confirms that CLSKA effectively expands the receptive field without increasing the parameter count. Overall, the Grad-CAM results align with the quantitative metrics, intuitively demonstrating the synergistic gains of the four improvements in feature discriminability and background robustness.

## Conclusion

This study addresses the numerous challenges faced in traffic sign detection tasks in autonomous driving scenarios by proposing a lightweight, high-precision detection algorithm based on an improved YOLOv11 model. The algorithm achieves this by innovatively incorporating the SPD-Conv module, NAST, CLSKA module, and NWD loss function, thereby significantly enhancing the detection accuracy and robustness of small traffic signs while maintaining model lightweightness. Through systematic experimental validation on the TT100K (Chinese urban road) dataset, the proposed algorithm demonstrates outstanding performance in terms of generalization capability across multiple scales, real-time processing, and small-object detection performance, providing an efficient and reliable solution for traffic sign detection tasks in autonomous driving systems.

In the course of our research, we first analyzed the limitations of traditional traffic sign detection methods and existing deep learning-based detection algorithms, particularly in terms of small object detection, adaptability to complex scenes, and model lightweighting. To address these issues, we proposed a series of innovative improvements. SPD-Conv rescues small-sign features during down-sampling; NAM re-uses BN scalars to mask clutter at zero cost; CLSKA widens receptive fields with separable large-kernel convolutions; NWD loss replaces IoU with Wasserstein distance to keep gradients alive for 8-pixel objects.

The experimental results show that the improved model achieves a 72.2 FPS increase in detection speed and a 2.0% improvement in accuracy, while reducing the number of parameters by approximately 17% and computational complexity by approximately 16.8%, significantly outperforming existing models. Particularly in small object detection, the improved model performs exceptionally well, effectively addressing the false negative issues encountered by traditional algorithms in detecting small objects at long distances. Additionally, through Grad-CAM heatmap visualization analysis, we can intuitively observe the significant improvements in feature discriminability and background robustness of the improved model, further validating the effectiveness of the various improvements.

Although this study has made some progress in the traffic sign detection task, there is still room for improvement. For example, in the future, we can further optimize the model structure and explore more efficient network architecture designs to further reduce the number of parameters and computational complexity of the model while maintaining or improving detection accuracy. Additionally, multimodal data fusion (such as the integration of lidar, millimeter-wave radar, and visual data) is an important direction for future research, which will further enhance the robustness and accuracy of traffic sign detection. In practical applications, it is also necessary to consider the model’s generalization capabilities across different countries, climatic conditions, and more complex scenarios to ensure its widespread application in autonomous driving systems.

## Discussion

YOLO-AML demonstrates a strong balance between lightweight design and high accuracy, making it well suited for real-time traffic sign detection in resource-limited environments. The integration of SPD-Conv, NAST, CLSKA, and NWD effectively addresses key challenges such as small object feature loss, background noise, and gradient vanishing. The model’s superior performance on the TT100K dataset highlights its robustness across complex scenes and scales.

Future work should explore quantization, pruning, or knowledge distillation to enhance efficiency. Moreover, incorporating multimodal inputs (e.g., LiDAR or thermal imaging) could improve detection under adverse weather or lighting conditions. Overall, YOLO-AML offers a promising direction for real-time, high-precision traffic sign detection, but continued refinement is essential for broader autonomous driving applications.

## Data Availability

The research data supporting the results of this manuscript are publicly available and can be accessed at the following URL: https://cg.cs.tsinghua.edu.cn/traffic-sign/tutorial.html
